# Chemical composition and biological activities of essential oils of two new chemotypes of Glebionis Cass.

**DOI:** 10.3906/kim-2104-11

**Published:** 2021-10-19

**Authors:** Hüseyin SERVİ

**Affiliations:** 1 Department of Pharmacognosy, Faculty of Pharmacy, Istanbul Yeni Yüzyıl University, İstanbul Turkey

**Keywords:** *Glebionis coronaria*, *Glebionis segetum*, essential oil, chemotype, antiinflammatory activity

## Abstract

This paper includes the result of the first study of the chemical composition, antioxidant, antiinflammatory, and antidiabetic activities of the essential oils of *Glebionis coronaria* (L.) Cass. ex Spach and *Glebionis segetum* (L.) Fourr. from Turkey. In the current study, nine and twenty-eight constituents were determined in the essential oils of aerial parts of *G*. *coronaria* (GCE) (92.1%) and *G*. *segetum* (GSE) (90.0%), respectively. The main components were capillin (65.9%) in GCE, capillene (53.4%) in GSE. The essential oil compositions were evaluated and compared with previous researches. In the current study, the plants are classified as chemotypes of *Glebionis* species. GCE and GSE showed poor and very poor DPPH radical scavenging activity, respectively. GCE and GSE exhibited significant and strong antiinflammatory activity against the 5-lipoxygenase enzyme, respectively. Also, GCE and GSE displayed moderate and weak antidiabetic activity against the α-glucosidase enzyme, respectively. Polyacetylenes were determined as the main class of compounds in GCE and GSE and had a notable antiinflammatory activity.

## 1. Introduction

The genus *Glebionis* Cass. is an annual herb and belongs to the Asteraceae family. *Glebionis* is extended in the Mediterranean, North Africa, Europe, and Asia. *Glebionis* is represented in Turkey by two species namely *Glebionis* *coronaria* (L.) Cass. ex Spach (syn.: *Chrysanthemum coronarium* L.) and *Glebionis segetum* (L.) Fourr. (syn.: *Chrysanthemum segetum* L.). *G*. *coronaria* leaves are 2-3-pinnatisect. The plant grows on banks, roadsides, and fields near the sea of East Thrace, West and South Anatolia region of Turkey. *G*. *segetum* leaves are 1-pinnatisect (or upper entire). The plant grows on fallow fields, cornfields, and roadsides of West and South Anatolia and North-West region of Turkey [1]. *G*. *coronaria* and *G*. *segetum* called as ‘Dağlama, Sarı papatya, Krizantem, Ale gömeci or Kasımpatı’ in Anatolia are traditionally used for the relief of abdominal pain and sore throat, treat shortness of breath and against hair loss by human [2]. They have antimicrobial, antioxidant, antiviral, antimycotic, cytotoxic activities [3–6]. *G*. *coronaria* is used as an edible plant in Japan and China. The leaves are used for the suppression of the fishy odors in food in Japan. Moreover, the leaves and stems of the Japanese sample have β-carotene, minerals, and vitamins in a high amount [7]. The aerial parts of both species are used as salad and food in the southern part of Turkey as well [8]. The essential oil constituents of *G*. *coronaria* have also been widely studied, whereas there is less report on the essential oil composition of *G*. *segetum*. The essential oils of *G*. *coronaria* displayed variation in their major compounds due to distinct geographical places. Camphor, α-pinene, lyratyl acetate, *trans*-chrysanthenyl acetate, *trans*-chrysanthenyl isovalerate, *cis*-chrysanthenyl acetate, *trans*-tonghaosu, α-humulene, γ-curcumene, (*Z*)-ocimene, myrcene, bornyl acetate, α-bisabolol, chrysanthemyl acetate, chrysanthemol, santolinatriene, and yomogi alcohol were found as main components of *G*. *coronaria* grown in Italy, Spain, Greece, Tunisia, Chile, South Korea, Ukraine, Jordan, and Cyprus [3,4,9–18]. (*E*,*E*)-α-farnesene, α-humulene, and tonghaosu were determined as the main compounds of *G*. *segetum* grown in Italy and China [4,19]. The previous studies displayed high variability in the essential oil ingredients and main compounds. In the current study, capillene and capillin were determined as the main compounds of *G*. *segetum* and *G*. *coronaria* collected from İstanbul, respectively. However, capillin and capillene were not determined in the essential oils of *G*. *coronaria* and *G*. *segetum* in previous studies [3,4,9–19]. There are no reports on the essential oil composition of *G*. *caronaria* and *G*. *segetum* from Turkey as well as on the α-glucosidase and 5-lipoxygenase inhibitory activity of GCE and GSE. This study aimed to specify the chemical composition and biological activities of the essential oils of two new chemotypes of *Glebionis*.

## 2. Materials and methods

### 2.1. Plant material

*G*. *coronaria* and *G*. *segetum* were collected in İkitelli-Başakşehir (41° 04’ 34.9’’ N; 28° 47’ 32.2’’ E) and Kayaşehir- Başakşehir (41° 7’ 36’’ N; 28° 46’ 53’’ N), İstanbul, Turkey in May 2017 and 2019 by Asst. Prof. Dr. Huseyin Servi and identified by Dr. Ahmet Dogan, respectively. Herbarium specimens of *G*. *coronaria* and *G*. *segetum* were deposited in the Marmara University Herbarium (Herbarium numbers: MARE20235 for *G*. *coronaria* and MARE22152 for *G*. *segetum*).

### 2.2. Distillation

The dried aerial parts of GCE (339 g) and GSE (420 g) were subjected to hydrodistillation for 3 h, using a Clevenger apparatus. 

### 2.3. Chromatographic analyses

The GC-MS analysis and determination of essential oils components were performed as described by Abu Zarga et al. [20]. In GC-MS analyses, the DP-5 (5% diphenyl, 95% dimethyl polysiloxane) capillary column (30m×0.25 mm, 0.25 m film thickness) and helium as carrier gas (0.9 mL/min) were used. The determination of the components was done by relative retention indices comparison of *n*-alkane series to the literature and with mass spectra comparison (Wiley 8th Ed./NIST 05 Mass Spectra library) [21].

The GC analyses were performed as described by Abu Zarga et al. [20]. The temperature of the FID detector was maintained at 300 °C. The same operating conditions and column were utilized in the GC-MS analyses.

### 2.4. DPPH radical scavenging activity

The free radical scavenging activity of the essential oils, based on the scavenging activity of the stable 1,1-diphenyl-2-picrylhydrazyl (DPPH) free radical was determined by the method described by Zou et al. [22]. Ascorbic acid (100-0.02 µg/mL) was used as a standard. In short, 10 µL of oils (250–0.49 µg/mL) or ascorbic acid dissolved in dimethylsulfoxide (DMSO) at different concentrations. In a 96-well plate, the oils and standard were mixed with 190 µL of 0.1 mM DPPH solution in MeOH. The reaction mixture was left in the dark at RT for 30 min. The absorbance of the mixture was measured spectrophotometrically at 517 nm. Each experiment was performed in triplicate. The percent radical scavenging activity of oils and standard against DPPH was calculated according to the following:

DPPH radical-scavenging activity (%) = [(A_0_–A_1_)/A_0_]×100

where A_0_ is the absorbance of the control (containing all reagents except the test compounds), and A_1_ is the absorbance of the oils/standard. Oil concentration providing 50% inhibition (IC_50_) was calculated from the graph plotting inhibition percentage against oil concentration.

### 2.5. In vitro antiinflammatory activity

5-lipoxygenase inhibition activity was analyzed by the method of Phosrithong and Nuchtavorn [23] with slight modifications described by Yildirim et al. [24]. Indomethacin (100–0.02 µg/mL) was used as a standard. 10 μL of oils (250–0.49 µg/mL) or indomethacin were added to ethanol (20 μL), pure water (20 μL), sodium borate buffer solution (25 μL, 0.1 M, pH 9) and type V soybean lipoxygenase solution (25 μL, 20.000 units/mL) in the buffer (pH 9). The reaction mixture was preincubated at 25 °C for 5 min. Then, linoleic acid solution (100 μL of 0.6 mM) was added to solutions, mixed well, and the change in absorbance at 234 nm was followed for 6 min. Each reaction was run in triplicate. The percent inhibition was calculated from the following equation:

% inhibition= [(A_control_−A_sample_)/A_control_]×100

A dose-response curve was plotted to determine the IC_50_ values. IC_50_ is defined as the concentration sufficient to obtain 50% of maximum antiinflammatory activity.

### 2.6. In vitro antidiabetic activity

The inhibition assay for α-glucosidase activity was conducted as described by Ramakrishna et al. [25] with slight modifications described by Sen et al. [26]. Acarbose (100–0.02 µg/mL) was used as a standard. In a 96-well plate, 10 µL of essential oils (250–0.49 µg/mL) or acarbose, 40 µL of 0.1 M sodium phosphate buffer (pH 6.9), and 100 µL of α-glucosidase (obtained from *Saccharomyces cerevisiae*) solution (1 unit/mL) were mixed. After preincubation (at 25 °C for 10 min), *p*-nitrophenyl-α-D-glucopyranoside (*p*NPG) (50 µL of 5 mM) to the solutions was added and reincubated at 25 °C for 5 min. The absorbance reading was taken before and after incubation at 405 nm using a microplate reader. Tests were performed in triplicate. The percentage inhibitory activity of the oils and standard against α-glucosidase enzyme were calculated according to the following:

α-glucosidase inhibitor activity (%) = [(A_0_–A_1_)/A_0_]×100

where A_0_ is the absorbance of the control (containing all reagents except the test compounds), and A_1_ is the absorbance of the oils/standard. Essential oils or standard concentration providing 50% inhibition (IC_50_) was calculated from the graph plotting inhibition percentage against oil or standard concentration.

### 2.7. Statistical analysis 

The results were indicated with ± standard deviation. The statistical analysis was done by Tukey’s Multiple Comparison Test with a confidence interval (CI) of 95% for each using the GraphPad Prism 5. Differences between means at p<0.05 levels were regarded as important.

## 3. Results and discussion

**Figure 1:**
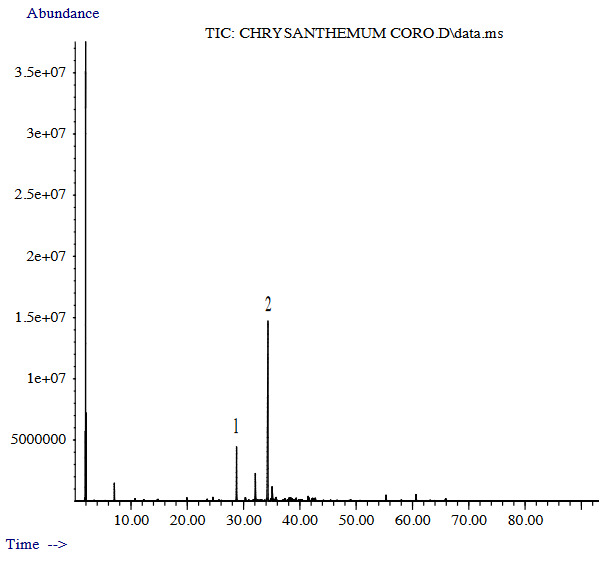
GC-MS chromatogram of the GCE (1: Capillene; 2: Capillin)

**Figure 2:**
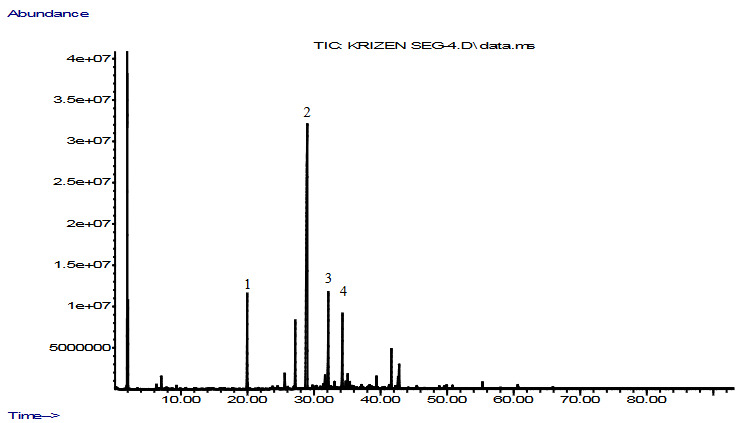
GC-MS chromatogram of the GSE (1: 1-Phenyl-penta-2,4-diyne; 2: Capillene; 3: Caryophyllene oxide; 4: Capillin)

The yields of GCE and GSE were 0.12 and 0.30 (v/w), respectively. Nine and twenty-eight constituents were determined in GCE (92.1%) and GSE (90.0%), respectively. The main components were determined as capillin 65.9% in GCE (given in Figure 1 and Table 1), and as capillene 53.4% in GSE (given in Figure 2 and Table 1). Other major compounds were capillene 11.9% and caryophyllene oxide 6.8% in the GCE, and caryophyllene oxide 9.2%, 1-phenyl-penta-2,4-diyne 7.3%, and capillin 7.2% in the GSE (Table 1). The GCE and the GSE were dominated by the presence of polyacetylenes (79.0% and 68.1%, respectively).

**Table 1:** The essential oil compositions of the aerial parts of G. coronaria and G. segetum.

RRI Exp.1	RRI Lit.2	Compounds	G. coronaria (%)	G. segetum (%)
932	939	α-Pinene	-	0.2
958	963	Benzaldehyde	2.6	0.6
1027	1029	Limonene	-	0.2
1284	1280	1-Phenyl-penta-2,4-diyne	0.4	7.3
1375	1377	α-Copaene	-	0.1
1419	1419	β-Caryophyllene	-	1.1
1453	1455	α-Humulene	-	0.2
1458	1458	(E)-β-farnesene	-	4.8
1496	1490	Capillene	11.9	53.4
1537	1546	1-(4-Methoxyphenyl)-2,4-pentadiyne	0.8	0.2
1552	1555	Isocaryophyllene oxide	-	0.2
1565	1563	(E)-Nerolidol	-	0.5
1571	1567	(Z)-3-hexenyl benzoate	-	1.0
1574	1574	Dendrolasin	-	0.3
1578	1574	Hexyl benzoate	-	0.6
1582	1581	Caryophyllene oxide	6.8	9.2
1585	1579	Isoaromadendrene epoxide	-	0.1
1593	1595	Salvial-4(14)-en-1-one	-	0.2
1609	1606	Humulene oxide II	-	0.5
1642	1637	Capillin	65.9	7.2
1668	1689	2,3-dihydro farnesol	-	0.1
1838	1837	Neophytadiene	-	0.3
1845	1847	Hexahydrofarnesyl acetone	0.9	-
1867	1868	Isobutyl-o-phthalate	-	0.3
2099	2086	9,12-15-octadecatrienoic acid methyl ester	-	0.2
2111	2116	Phytol	-	0.2
2298	2300	Tricosane	1.3	0.5
2498	2500	Pentacosane	1.5	0.3
		Phenyl alkynes	79.0	68.1
		Monoterpenes	-	0.4
		Sesquiterpenes	7.7	17.0
		Diterpenes	-	0.2
		Fatty acid derivatives	2.8	1.1
		Others	2.6	3.1
		Total identified compounds	92.1	90.0

1RRI Exp.: Relative retention indices calculated against n-alkanes (C5-C30); 2RRI Lit: Relative retention indices given in the literature for the compound in similar columns and analysis conditions.

The essential oils of *G*. *coronaria* and *G*. *segetum* possessed remarkable diversities in the main compounds. These diversities could be related to morphological characteristics and different geographical origins. The main compounds of the essential oil from flowers of *G*. *coronaria* grown in Sardinia-Italy were α-humulene, camphor, and γ-curcumene [4]; camphor and *cis*-chrysanthenyl acetate from a population in Tuscany-Italy [13]; *trans*-tonghaosu from populations in Sicily and Campania-Italy [16]; camphor, α-pinene and lyratyl acetate from a population Murcia-Spain [10]; *trans*-chrysanthenyl acetate and *trans*-chrysanthenyl isovalerate from a population in Korinthos-Greece [12]; camphor from a population in Attiki-Greece [12]; *cis*-chrysanthenyl acetate and *trans*-chrysanthenyl acetate from a population in Zanghouan-Tunisia [14]; camphor, *cis*-chrysanthenyl acetate and bornyl acetate from a population in Chile [15]; chrysanthemyl acetate and chrysanthemol from a population in Ukraine [3]; camphor, perilla aldehyde and *cis*-chrysanthenyl acetate from a population in Jordan [17]. The main compounds of essential oil from stems and leaves of *G*. *coronaria* were myrcene, α-bisabolol and (*E*,*E*)-α-farnesene from a population in Namyamju-South Korea [18]; (*Z*)-ocimene and myrcene from a population in Sicily-Italy [16]. The main compounds of the essential oil from aerial parts of *G*. *coronaria* were myrcene and (*E*)-β-farnesene from a population in Jordan [17]; camphor, santolinatriene, yomogi alcohol, *cis*-chrysanthenyl acetate, and bornyl acetate from a population in Cyprus [9]. The main compounds of the essential oil from flowers of *G*. *segetum* were (*E*,*E*)-α-farnesene and α-humulene from a population in Sardinia-Italy [4]; tonghaosu from a population in China [19]. These main compounds were found either in low concentration or not determined in the oils of the present study. The absence of these main compounds in the present study indicates the different chemovarieties of the plants. The previous reports displayed that *G*. *coronaria* and *G*. *segetum* collected from Italy, Spain, Greece, Tunisia, Chile, South Korea, Ukraine, Jordan, China, and Cyprus had monoterpenes, oxygenated monoterpenes, sesquiterpenes, oxygenated sesquiterpenes, and acetylenes as main groups. In the present research, GCE and GSE have polyacetylenes as the main group and displayed dissimilar chemical profiles from the previous studies.

The variations of the essential oil ingredients and composition may be connected to factors such as plant parts used, geographical regions, genotype, ecotype, chemotype, phenophases, and the environmental factors which can be temperature differences, relative humidity, irradiance, and photoperiod. The quantitative composition of the volatile oils of numerous aromatic plants is significantly influenced by the harvesting time, plant age, and product density [27].

Capillene and capillin were mainly determined from the essential oil of various *Artemisia* species including *A*. *campestris* [28], *A*. *campestris* var. *glutinosa* [29], *Artemisia* *capillaris* [30], *A*. *dracunculus* [31], *A*. *glauca* [32], *A*. *lehmanniana* [31], *A*. *monosperma* [33], *A*. *ordosica* [34], *A*. *scoparia* [35], *A*. *stricta* [36], and *Santolina rosmarinifolia* L. ssp. *rosmarinifolia* [37].

Capillene and capillin are polyacetylenes that are a group of bioactive secondary metabolites. Polyacetylenes display toxic features that possess strong skin sensitization, irritant, and allergic contact dermatitis, and neurotoxicity at high concentrations. Despite the toxic properties of polyacetylenes, many studies reported that polyacetylenes have various biological activities such as antidiabetic, antitumor, antiinflammatory, and antimicrobial activities [38-41]. Additionally, capillene and capillin displayed significant antimicrobial, antiapoptotic, cytotoxic, anticancer, antifeedant, and antiinflammatory activities [42-44]. 

There are studies on DPPH radical scavenging activity of essential oil of *Chrysanthemum coronarium* (*Glebionis coronaria*) in the literature. In a study, Polatoglu et al. reported that *C*. *coronarium* (*G*. *coronaria*) essential oil did not have significant DPPH radical scavenging activity [9]. In another study, Hosni et al. found that hydro-distilled essential oil obtained from the *C*. *coronarium* flowerheads had DPPH radical inhibition lesser than 15% at a concentration of 200 µg/mL [14]. However, there is no study on the DPPH radical scavenging activity of the essential oil of *Glebionis segetum*. In the current study, GCE and GSE showed poor and very poor radical scavenging activity with IC_50_ values of 1.020 and 2.525 mg/mL against DPPH radical, respectively (given in Table 2).

**Table 2:** Antioxidant, antiinflammatory, and antidiabetic activities of the GCE and GSE.

Essential oilsand standards	DPPH radical scavenging activity	Antilipoxygenaseactivity	α-glucosidase inhibitory activity
	IC50 (mg/mL)		
GCE*	1.020 ± 0.004b	0.151 ± 0.001c	0.499 ± 0.006b
GSE*	2.525 ± 0.017c	0.017 ± 0.003a	0.967 ±0.006c
Ascorbic acid	0.018 ± 0.000a		
Indomethacin		0.022 ± 0.000b	
Acarbose			0.040 ± 0.002a

*Abbreviations: GCE and GSE show essential oils of aerial parts of Glebionis coronaria and Glebionis segetum, respectively. **Each value in the table is represented as mean ± SD (n = 3). Different letter superscripts in the same column indicate significant differences (p < 0.05).

GCE and GSE displayed moderate and weak antidiabetic activity with IC_50_ values of 0.499 and 0.967 mg/mL against the α-glucosidase enzyme, respectively (given in Table 2). There is no study on the α-glucosidase inhibitory activity of essential oils of GCE and GSE in the literature. However, there is a study on the α-glucosidase inhibitory activity of capillin obtained from CH_2_Cl_2_ fraction of MeOH extract of *A*. *capillaris*. Capillin showed strong α-glucosidase inhibitory activity [38]. Thus, capillin, which was the main compound of GCE may be responsible for the antidiabetic activity of the oil.

Also, the GCE and GSE exhibited significant and strong antiinflammatory activity with IC_50_ values of 0.151 and 0.017 mg/mL against 5-lipoxygenase enzyme, respectively (given in Table 2). Although there is no report on the 5-lipoxygenase enzyme inhibitory or antiinflammatory activity of GCE and GSE, there are studies on the antiinflammatory activity of the extracts of these species. In a study, Strzelecka et al. notified that ethanol extract of *C*. *coronarium* (*G*. *coronaria*) decreased cytokine or LPS-stimulated iNOS mRNA levels in MBE (Murine brain microvascular endothelial cells) and P388D1 (Murine monocyte/macrophage-like cell line cells) [45]. In another study by Mascolo et al. found the ethanol extract of *C*. *segetum* (*G*. *segetum*) inhibited carrageenin foot edema by 11% in rats (100 mg/kg p.o.) [46]. Capillin and capillene have been notified to have antiinflammatory features in previous studies [47,48]. Thus, capillin and capillene, determined as main compounds in the GCE and GSE may be responsible for the antiinflammatory activity of the oils.

## 4. Conclusion

In general, the essential oil composition of the current study showed differences in quality and quantity from the previous research. These differences of the current study may be considered as chemotypes, which can be named capillin and capillene chemotypes. As a result, polyacetylenes were the main group of the GCE and GSE and had a notable antiinflammatory activity. However, in vivo studies are required to verify our findings.

## References

[ref1] Davis PH 1982 Flora of Turkey and the East Aegean Islands

[ref2] Tuzlacı E. Türkiye Bitkileri Geleneksel İlaç Rehberi 2016 Turkey: İstanbul Tıp Kitabevleri

[ref3] Ivashchenko IV 2017 Chemical composition of essential oil and antimicrobial properties of Chrysantemum coronarium (Asteraceae) Biosystems Diversity 25 119 123

[ref4] Marongiu B Piras A Porcedda S Tuveri E Laconi S 2009 Chemical and biological comparisons on supercritical extracts of Tanacetum cinerariifolium (Trevir) Sch. Bip. with three related species of chrysanthemums of Sardinia (Italy) Natural Product Research 23 190 199 19173127 10.1080/14786410801946221

[ref5] Derouiche K Zellagui A Gherraf N Belbout A 2018 Evaluation of antioxidant and antimicrobial activities of flowers extracts of Chrysanthemum segetum L World 7 106 111

[ref6] Kim J Choi JN Ku KM Kang D Kim JS 2011 A correlation between antioxidant activity and metabolite release during the blanching of Chrysanthemum coronarium L Biochemistry 75 674 680 10.1271/bbb.100799 21512247

[ref7] DJ Mabberley 1997 The Plant Book

[ref8] Tuzlacı E. Türkiye 2011 ’nin yabani besin bitkileri ve ot yemekleri

[ref9] Polatoğlu K Karakoç OC Demirci B Başer KHC 2018 Chemical composition and insecticidal activity of edible garland (Chrysanthemum coronarium L.) essential oil against the granary pest Sitophilus granarius L. (Coleoptera) Journal of Essential Oil Research 30 120 130

[ref10] Alvarez-Castellanos PP Bishop CD Pascual-Villalobos MJ 2001 Antifungal activity of the essential oil of flowerheads of garland chrysanthemum (Chrysanthemum coronarium) against agricultural pathogens Phytochemistry 57 99 102 11336267 10.1016/s0031-9422(00)00461-1

[ref11] Alvarez-Castellanos PP Pascual-Villalobos MJ 2003 Effect of fertilizer on yield and composition of flowerhead essential oil of Chrysanthemum coronarium (Asteraceae) cultivated in Spain Industrial Crops and Products 17 77 81

[ref12] Basta A Pavlović M Couladis M Tzakou O 2007 Essential oil composition of the flowerheads of Chrysanthemum coronarium L. from Greece Flavour and Fragrance Journal 22 197 200

[ref13] Flamini G Cioni PL Morelli I. 2003 Differences in the fragrances of pollen, leaves, and floral parts of garland (Chrysanthemum coronarium) and composition of the essential oils from flowerheads and leaves Journal of Agricultural and Food Chemistry 51 2267 2271 12670168 10.1021/jf021050l

[ref14] Hosni K Hassen I Sebei H Casabianca H 2013 Secondary metabolites from Chrysanthemum coronarium (Garland) flowerheads: Chemical composition and biological activities Industrial Crops and Products 44 263 271

[ref15] Sebastián B Urzúa AM Vines M. 2006 Analysis of surface and volatile compounds of flower heads of introduced plants of Chrysanthemum coronarium L. growing wild in Chile Flavour and Fragrance Journal 21 783 785

[ref16] Senatore F Rigano D De Fusco R Bruno M. 2004 Composition of the essential oil from ﬂowerheads of Chrysanthemum coronarium L. (Asteraceae) growing wild in Southern Italy Flavour and Fragrance Journal 19 149 152

[ref17] Tawaha K Hudaib M. 2010 Volatile oil profiles of the aerial parts of Jordanian garland, Chrysanthemum coronarium Pharmaceutical Biology 48 1108 1114 20818927 10.3109/13880200903505641

[ref18] Zheng CH Kim TH Kim KH Leem YH Lee HJ 2004 Characterization of potent aroma compounds in Chrysanthemum coronarium L. (Garland) using aroma extract dilution analysis Flavour and Fragrance Journal 19 401 405

[ref19] Wu ZH Wang J Li JC Xu YZ Yu AL 1994 Antifeeding activity and chemical composition of the essential oil from Chrysanthemum segetum L Natural Product Research and Development 6 1 4

[ref20] Abu Zarga MH Al-Jaber HI Baba Amer ZY Sakhrib L Al-Qudah MA 2013 Chemical composition, antimicrobial and antitumor activities of essential oil of Ammodaucus leucotrichus growing in Algeria Journal of Biologically Active Products from Nature 3 224 231

[ref21] 2006

[ref22] Zou Y Chang SK Gu Y Qian SY 2011 Antioxidant activity and phenolic compositions of lentil (Lens culinaris var. morton) extract and its fractions Journal of Agricultural and Food Chemistry 59 2268 2276 21332205 10.1021/jf104640k PMC3063125

[ref23] Phosrithong N Nuchtavorn N. 2016 Antioxidant and antiinflammatory activites of Clerodendrum leaf extracts collected in Thailand European Journal of Integrative Medicine 8 281 285

[ref24] Yıldırım A Şen A Doğan A Bitis L 2019 Antioxidant and antiinflammatory activity of capitula, leaf and stem extracts of Tanacetum cilicicum (Boiss International Journal of Secondary Metabolite 6 211 222

[ref25] Ramakrishna R Sarkar D Schwarz P Shetty K. 2017 Phenolic linked antihyperglycemic bioactives of barley (Hordeum vulgare L.) cultivars as nutraceuticals targeting type 2 diabetes Industrial Crops and Products 107 509 517

[ref26] Sen A Kurkcuoglu M Senkardes I Bitis L Baser KHC 2019 Chemical composition, antidiabetic, antiinflammatory and antioxidant activity of Inula ensifolia L. essential oil Journal of Essential Oil Bearing Plants 22 1048 1057

[ref27] Chauhan RS Kitchlu S Ram G Kaul MK Tava A North-West Himalaya 2010 Chemical composition of capillene chemotype of Artemisia dracunculus L. from Industrial Crops and Products 31 546 549

[ref28] Erel SB Reznicek G Şenol SG Yavaşoğlu NUK Konyalioğlu S 2012 Antimicrobial and antioxidant properties of Artemisia L. species from western Anatolia Turkish Journal of Biology 36 75 84

[ref29] Juteau F Masotti V Bessière JM Viano J 2002 Compositional characteristics of the essential oil of Artemisia campestris var glutinosa. Biochemical Systematics and Ecology 30 1065 1070

[ref30] Cha JD Jeong MR Jeong SI Moon SE Kim JY 2005 Chemical composition and antimicrobial activity of the essential oils of Artemisia scoparia and A. capillaris Planta Medica 71 186 190 15729631 10.1055/s-2005-837790

[ref31] Mohammadhosseini M. 2017 Essential oils extracted using microwave-assisted hydrodistillation from aerial parts of eleven Artemisia species: Chemical compositions and diversities in different geographical regions of Iran Records of Natural Products 11 114 129

[ref32] Polyanskaya EV Korolyuk EA Tkachev AV 2007 Composition of essential oil from Artemisia glauca from western Siberia Chemistry of Natural Compounds 43 544 547

[ref33] Badawy ME Abdelgaleil SA 2014 Composition and antimicrobial activity of essential oils isolated from Egyptian plants against plant pathogenic bacteria and fungi Industrial Crops and Products 52 776 782

[ref34] Fenglan Y Maohua M Lingshao K. 1996 Chemical compositions of essential oil from stems and leaves of Artemisia ordosica in Maowusu sandy Mongolia Natural Product Research and Development 8 14 18

[ref35] Ickovski JD Stepić KD Stojanović GS 2020 Composition of essential oils and headspace constituents of Artemisia annua L. and A. scoparia Waldst Journal of the Serbian Chemical Society 85 1565 1575

[ref36] Manika N Chanotiya CS Darokar M Singh S Bagchi GD 2016 Compositional characters and antimicrobial potential of Artemisia stricta Edgew. f. stricta Pamp. essential oil Records of Natural Products 10 40 46

[ref37] Palá-Paúl J Pérez-Alonso MJ Velasco-Negueruela A Ramos-Vázquez P Gómez-Contreras F 1999 Essential oil of Santolina rosmarinifolia L. ssp. rosmarinifolia: first isolation of capillene, a diacetylene derivative Flavour and Fragrance Journal 14 131 134

[ref38] Islam MN Choi RJ Jung HA Oh SH Choi JS 2016 Promising antidiabetic potential of capillin and capillinol isolated from Artemisia capillaris Archives of Pharmacal Research 39 340 349 26832324 10.1007/s12272-016-0715-y

[ref39] Sugimoto N Tada A Yamazaki T Tanamoto K. 2007 Antimicrobial activity and constituents in rumput roman extract as a natural food preservative Journal of the Food Hygienic Society of Japan 48 106 111 17892004 10.3358/shokueishi.48.106

[ref40] Hallock YF Cardellina JH Balaschak MS Alexander MR Prather TR 1995 Antitumor activity and stereochemistry of acetylenic alcohols from the sponge Cribrochalina vasculum Journal of Natural Products 58 1801 1807 8691203 10.1021/np50126a001

[ref41] Shim H Moon JS Lee S Yim D Kang TJ 2012 Polyacetylene compound from Cirsium japonicum var. ussuriense inhibited caspase-1-mediated IL-1β expression Immune Network 12 213 216 23213315 10.4110/in.2012.12.5.213 PMC3509166

[ref42] Whelan LC Ryan MF 2004 Effects of the polyacetylene capillin on human tumour cell lines Anticancer Research 24 2281 2286 15330173

[ref43] Masuda Y Asada K Satoh R Takada K Kitajima J. Capillin 2015 a major constituent of Artemisia capillaris Thunb. flower essential oil induces apoptosis through the mitochondrial pathway in human leukemia HL-60 cells Phytomedicine 22 545 552 25981920 10.1016/j.phymed.2015.03.008

[ref44] Yano K. 1983 Insect antifeeding phenylacetylenes from growing buds of Artemisia capillaris Journal of Agricultural and Food Chemistry 31 667 668

[ref45] Strzelecka M Bzowska M Koziel J Szuba B Dubiel O 2005 Antiinflammatory effects of extracts from some traditional Mediterranean diet plants Journal of Physiology and Pharmacology Supplement 56 139 156 15800391

[ref46] Mascolo N Autore G Capasso F Menghini A Fasulo MP 1987 Biological screening of Italian medicinal plants for antiinflammatory activity Phytotherapy Research 1 28 31

[ref47] Abdul QA Seong SH Ahn BR Islam MN Jung HA 2018 Antiinflammatory potential of Artemisia capillaris and its constituents in LPS-induced RAW264. 7 Cells Natural Product Sciences 24 171 180

[ref48] Cai H Song YH Xia WJ Jin MW 2006 Aqueous extract of Yin-Chen-Hao decoction, a traditional Chinese prescription, exerts protective effects on concanavalin A-induced hepatitis in mice through inhibition of NF-kB Journal of Pharmacy and Pharmacology 58 677 684 16640837 10.1211/jpp.58.5.0013

